# The biomechanics of lower limb injuries in frontal-impact road traffic collisions

**DOI:** 10.4314/ahs.v18i2.17

**Published:** 2018-06

**Authors:** Mohannad B Ammori, Fikri M Abu-Zidan

**Affiliations:** 1 School of Engineering, University of Cardiff, United Kingdom; and Royal Gwent Hospital, Newport, United Kingdom; 2 Department of Surgery, College of Medicine and Health Sciences, UAE University,17666 Al-Ain, United Arab Emirates

**Keywords:** Biomechanics, frontal-impact collisions, lower limb injury, knee, thigh and hip injury, lower leg, foot and ankle injury

## Abstract

**Aim:**

We aimed to review the biomechanics of lower limb injuries caused by frontal-impact road traffic collisions.

**Methods:**

In this narrative review, we identified articles through pubmed, Scopus and Science Direct search engines for the period of 1990–2014. Search terms included: “biomechanics”, “lower limb injury”, “hip injury”, “knee injury”, “foot and ankle injury” and “frontal impact collision”. We studied factors affecting the anatomical site, frequency and severity of the injuries.

**Results:**

The most common reported mechanisms of injury were: the impaction of the knee with the dashboard resulting in acetabular fracture or posterior hip dislocation; and toepan intrusion in combination with forceful application of the brake resulting in foot and ankle fractures. The probability of an occupant sustaining significant injury to the hip is increased in taller males, and being out of position during the collision. The probability of an occupant sustaining a fracture to the foot and ankle is increased in shorter female occupants with a large overlap impact or a near oblique collision.

**Conclusion:**

Understanding the biomechanics of frontal-impact road traffic collisions is useful in alerting clinicians to the potential lower limb injuries sustained in these collisions.

## Introduction

The severity and distribution of lower limb injuries sustained in frontal-impact road traffic collisions are dependent on different factors. Understanding the biomechanics of these injuries at the point of impact and their contributing factors will help us to diagnose them. Frontal-impact collisions may result in acetabular fractures or posterior hip dislocations following the impact of the knee against the dashboard.^1^ In contrast, injuries to the foot and ankle result from compartment intrusion.^2^ The contraction of the muscles of the lower limb during the forceful application of the brake generates internal and external compressive forces, increasing the risk of foot and ankle injuries.^3^ We aimed to review the literature on the biomechanics of the lower limb injuries sustained during a frontal-impact collision, so as determine the factors predictive of the site, frequency and severity of these injuries.

## Methods

Pubmed, Scopus and Science Direct search engines were searched for the time period 1990–2014. Articles were identified through the electronic databases. Hand search of the references of the retrieved published articles were then performed. The original search terms were constructed from the primary concepts of this literature review, which included the following: “biomechanics”, “lower limb injury”, “hip injury”, “knee injury”, “foot and ankle injury” and “frontal impact collision”. Studies were included in the review provided the following criteria were met: a) the study was written in English, b) the study included only human adult subjects, c) the direction of impact was frontal in the majority, and d) lower limb injuries were considered in population-based studies. The included studies were experimental studies using computational simulations, biomechanical studies using volunteers, crash test dummies or cadavers, and population studies.

Studied excluded were 1) those which investigated injuries to children, motorcyclists or pedestrians, and 2) those which did not primarily study the biomechanics of the lower limb in frontal impact collisions. This study is a narrative review in which the strict rules of systematic reviews of following a precise protocol and search were not followed.

## Results

Thirty papers were included in this review. They were from the USA^4^–^25^, UK^26^,^27^, Australia^28^,^29^, France^3^,^30^, Germany^31^ and Sweden^2^. The most frequently sampled databases throughout the literature were the Crash Injury Research and Engineering Network (CIREN), the National Automobile Sampling System (NASS) and the Crashworthiness Data System (CDS).

### Occupant's demographics

**Fig 1:**
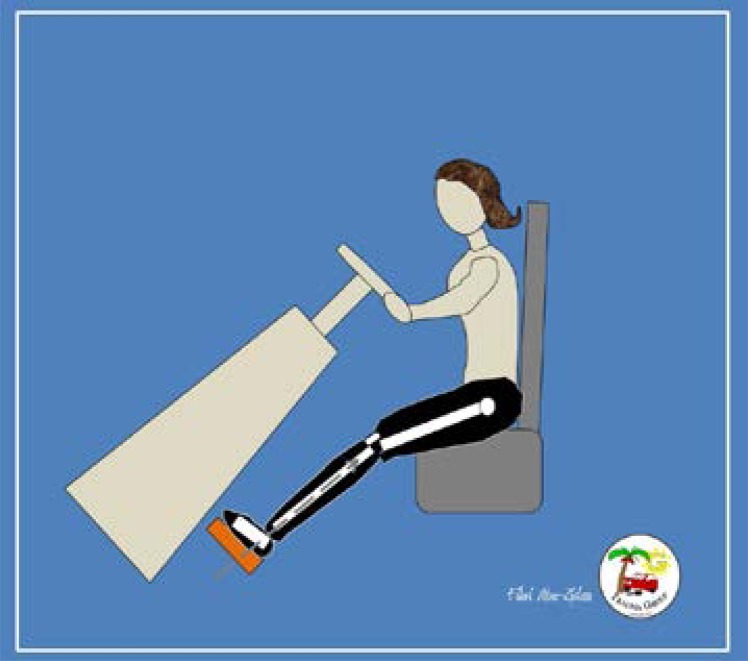
Males are usually taller than females. This increases their chance of having Knee-thigh-hip injuries (dashed arrow) when the knee directly hits the dashboard.

**Fig 2:**
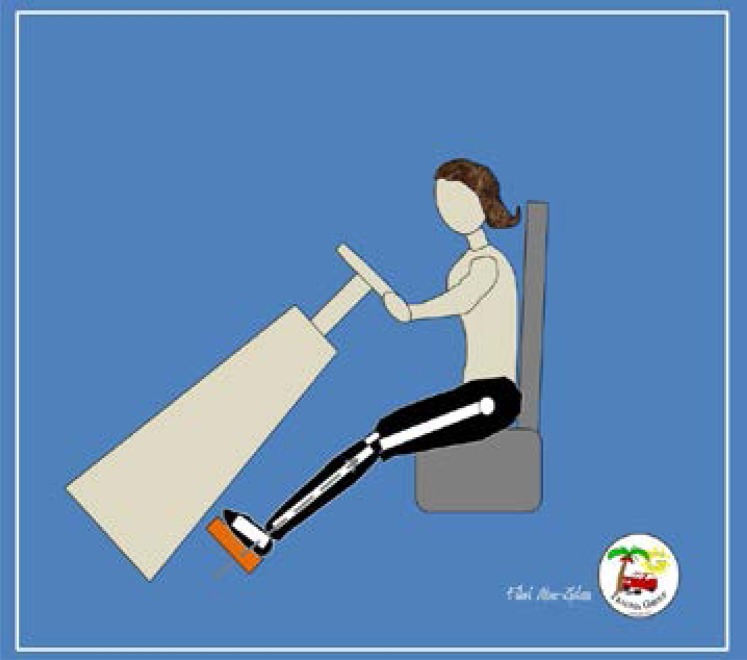
Several studies have reported a higher incidence of below-knee injuries in shorter female vehicle occupants. They may need to extend their knee to reach the pedal. The direction of frontal impact will involve the foot and lower leg (dashed arrow).

Occupant variables defining the nature of lower limb injury included gender, height, posture, age and weight of the occupant. The probability of an occupant sustaining significant injury to the hip is increased in taller males, and being out of position during the collision. The probability of an occupant sustaining a fracture to the foot and ankle is increased in shorter female occupants with a large overlap impact or a near oblique collision.^4^–^7^ Figures 1 and 2 explain the difference of biomechanics of lower limb injuries in males and females in frontal impact collisions. Obesity was identified as a risk factor for lower limb injury, especially femoral fracture.^10^–^11^ Elderly females are at an increased risk of sustaining a fracture during a motor vehicle collision.^26^,^28^

### Impact and vehicle

The design of the vehicle, the energy transmitted on impact and the extent of vehicle overlap in frontal-impact collisions are important predictive variables of the incidence and distribution of injuries in the lower limb. The most common reported mechanisms of injury were: the impaction of the knee with the dashboard resulting in acetabular fracture or posterior hip dislocation; and toepan intrusion in combination with forceful application of the brake resulting in foot and ankle fractures.^7^,^22^,^27^,^29^

### Injuries sustained

The tolerance of the hip joint is important in defining the nature of injuries sustained in frontal-impact collisions. Direct axial femoral forces increase the risk of hip fractures. 13–15 During a frontal-impact collision, the forward motion of the occupant in combination with the intrusion of the pedal could subject the ankle to dorsiflexion and the application of direct external loads. The out of position femur during the application of the brake with the right lower limb would result in dashboard impaction of the knee. Encapsulation of the knee or the foot may produce fractures of tibia and fibula due to torsional forces.^7^,^21^,^22^,^29^

## Discussion

Several studies have reported a higher incidence of below-knee injuries in shorter female vehicle occupants.^4^–^7^ Dischinger et al reported a higher incidence of lower extremity fractures in shorter adults, the majority of which were foot and ankle injuries in female occupants.^4^ Chong et al reported that female occupants sustained a higher proportion of open foot and ankle fractures compared with male occupants, who suffered from a higher proportion of closed knee-thigh-hip fractures. The authors thought that this discrepancy was attributed to the height of the vehicle occupants, and not the gender because male occupants were significantly taller than the females.^5^ The same findings were reported from the CIREN database, as short stature was identified as a positive predictor of tibial fracture.^6^ Sochor et al also reported a higher incidence of hip injuries in taller, heavier male occupants, but acknowledged gender as an additional variable explaining this injury.^7^ The acetabular cup of the female pelvis is orientated to a lesser extent in the horizontal and lateral directions compared with the male pelvis. This provides a greater reactive surface for femoral loading. Furthermore, a greater acetabular depth and a lesser diameter of the femoral head enhance stability of the female hip when subjected to femoral stress in frontal-impact collisions. This reduces the risk of injury to the hip, and increases the risk of injury to the thigh or the knee.^1^ Differences in the injuries sustained between vehicle occupants of different genders may also be attributed to differences in the angle of the ankle relative to the femur or tibia depending on the seating position.^1^,^4^ A study using numerical simulations in conjunction with established injury risk functions reported that posture of the vehicle occupant is the best indicator of lower limb injury in frontal-impact collisions.^9^

Although age was not identified as a predictive variable of femoral or tibial fractures from the CIREN database^6^, a recent study of data sampled from the NASS and CDS have found an age of ≤ 17 and the male sex to be associated with a lower incidence of lower extremity injury in comparison to the general cohort.^25^ In a separate study, female vehicle occupants and those above the age of 60 were reported to sustain injuries at a collision having less energy transmission than the general population.^26^ Elderly females are at an increased risk of sustaining a fracture as a result of the development of osteoporosis following menopause.^28^ Since energy = ½ (mass × velocity^2^), a greater occupants mass is expected to potentiate the energy generated upon impact. In addition to this, obese patients had a greater forward excursion of the knee and pelvis.^10^,^11^ This may explain the higher incidence of lower limb injury in obese occupants.

There is a direct relationship between the incidence of lower limb injury and the velocity of impact.^7^,^29^ The overlap, or the distribution of damage across the front of the vehicle, influences the risk of injury, with a greater incidence of injuries to the lower extremities in large overlap frontal-impact collisions. However, a small overlap was more frequently associated with severe injuries to the lower limb. This can be explained by the lower frequency of mild injuries such as tarsal or metatarsal fractures in small overlap collisions because a higher proportion of vehicle occupants were subjected to forces transmitted throughout the knee-thigh-hip region as opposed to the foot and ankle. The authors attributed this to the anatomical positioning of the occupant and the oblique point of application of stress to the lower limb.^12^

**Figure 3:**
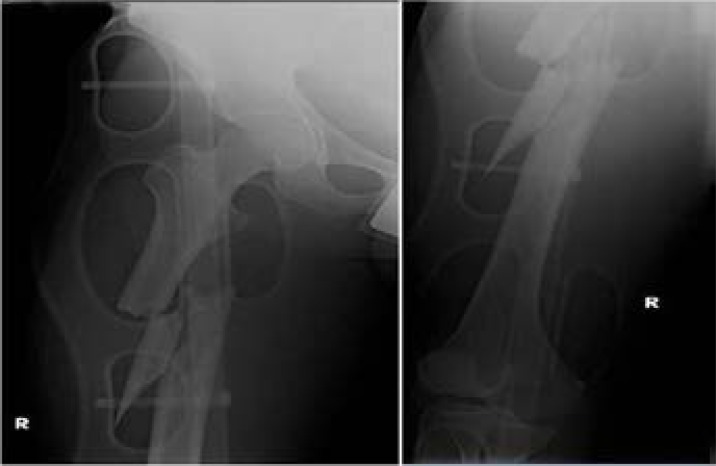
25-year-old male driver travelling at a high speed (100 km/hour) was involved in a front impact collision. The patient was not wearing a seatbelt but the airbag was released. The patient was spared from having head or chest injury but sustained a comminuted mid-diaphyseal femoral fracture.

The safety of occupants is affected by the size, design and age of the vehicle. Sixteen percent of drivers in large vehicles suffered from moderate injuries compared with 26% of drivers in small vehicles in frontal-impact collisions. This difference was not observed for injuries to the lower limb.^27^ The development of safety in motor vehicles has led to a reduction in the risk of occupant injury in frontal-impact collisions. Page et al reported a decrease in the incidence and severity of the injuries sustained by restrained occupants in newer vehicles, with a close to 50% reduction in the frequency of severe injuries.^30^ The advent of air bag deployment has resulted in an increase in the number of occupants whose injury of greatest severity is contained within the lower limb as opposed to a more critical region.^7^ (Figure 3). The deployment of knee air bags prevents occupants from sliding out from underneath the lap belt through preventing forward motion of the lower limbs.^30^

Rupp et al evaluated the tolerance of the human hip in unembalmed cadavers, when subjected to a dynamic load applied to the knee along the long axis of the femur. They reported a higher tolerance of the femoral neck compared with the acetabulum (7.59 kn compared with 5.70 kn, respectively).^13^ For an average size male crash test dummy, a 50% risk of sustaining a hip fracture occurred with direct axial femoral forces of 6.73 kn.^14^ However, in a study of relatively low speed frontal collisions, a discrepancy was observed between the estimated axial load and the expected severity of injuries sustained by the vehicle occupants. The authors suggested that the occupants' femurs have been subjected to additional axial loading, as a result of compressive forces generated by the contraction of muscles whilst bracing for impact. To identify the additional compressive forces generated by the application of the brake, the authors calculated the mean maximum extensor muscle torque around the knee. This produced an additional compressive force of 5.38 kn and 3.35 kn for males and females, respectively. Furthermore, the mean maximum flexor muscle torques generated an additional 3.00 kn for males and 1.80 kn for females.^15^ The tolerance of the hip is influenced by the posture^16^, and therefore it is important to note a reported 96° of right hip flexion during the application of the brake.^3^ A reduction of 4% of the tolerance of the hip was reported with 30° of flexion and by 8% with 10° of adduction from the typical or neutral posture of the hip for a seated driver.^16^ These results would suggest that out of position occupants were at an increased risk of hip fracture. However, the relative risk of fractures of other components of the lower limb was not evaluated.

**Figure 4:**
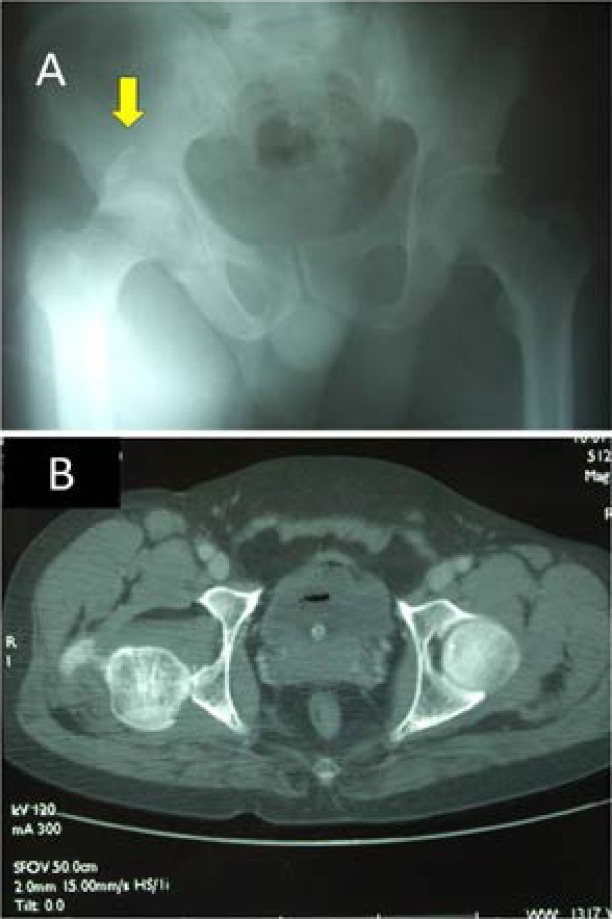
30-year-old male front-seat passenger was involved in a frontal impact road traffic collision. He sustained a posterior dislocation of the right hip, fracture of the posterior rim of the right acetabulum (yellow arrow) **(A, B)** and a right intra-capsular neck of femur fracture. Notice the lateral orientation of the acetabulum which is shown in the CT scan **(B)**.

Hallman et al reported that inter-trochanteric fractures were the most common type of hip fracture in small overlap collisions.^12^ In contrast Rupp et al reported that tolerance was high within the diaphysis and the distal femur, while the posterior acetabulum was the weakest component.^13^ Wang et al demonstrated that the nature of lower limb injury is influenced by the orientation of the acetabulum. In the laterally oriented acetabulum, the force applied from the femur will affect the posterior edge of the socket, resulting in increased acetabular fractures and posterior dislocation (Figure 4). However, in the anteriorly oriented acetabulum, the femoral load will affect a greater surface area, reducing acetabular fractures and increasing fractures of the femur or the knee.^1^

**Figure 5:**
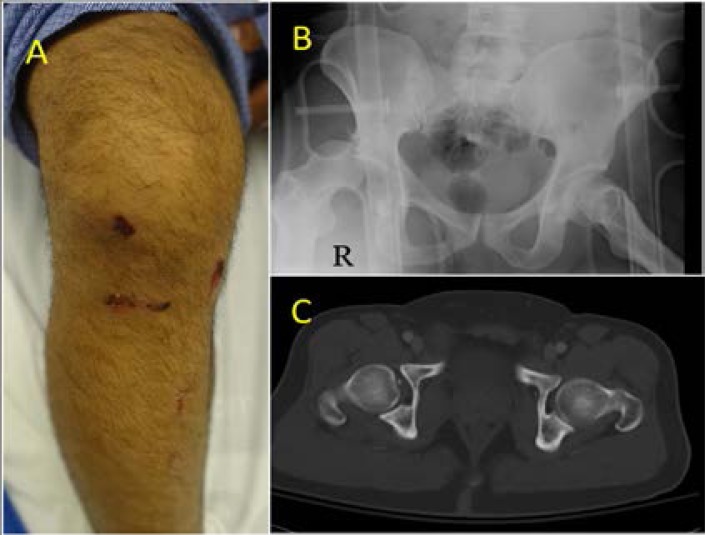
42-year-old male driver had a frontal impact collision presented complaining of the right knee and hip pain. The right hip was internally rotated with flexion (60 degree). The right knee was swollen having multiple abrasions **(A).** Pelvic X-ray showed posterior dislocation of the right hip **(B).** Post reduction CT scan showed fracture of posterior rim of the acetabulum and widened hip joint space **(C)**.

Knee impaction and compartment intrusion could result in injuries to the patella and the long bones of the lower limb.^17^ Impaction with the dashboard or the steering wheel was specifically associated with knee injury.^29^ Furthermore, intrusion into the passenger compartment resulted in severe injury to the knee.^31^ Studies have demonstrated a positive correlation between the incidence of knee injury and the velocity of the vehicle at the time of impact.^18^,^31^ Interestingly, a higher impact velocity was needed to result in ligamentous injuries compared with fractures around the knee. This was attributed to direct impact forces. Ligamentous injuries were thought to result from indirect forces through the femur or thigh. The association between knee injury and acetabular fracture or posterior dislocation of the hip was rare as reported by a study on restrained vehicle occupants.^31^ The authorsfound that the classical triad of dashboard injuries described as a mid-diaphyseal femoral fracture, ipsilateral hip injury and disruption of the extensor mechanism of the knee was observed only in 5.8% (n=5) of 82 patients who had an Abbreviated Injury Scale ≥2 injuries of the knee.^31^ (Figure 5).

**Figure 6:**
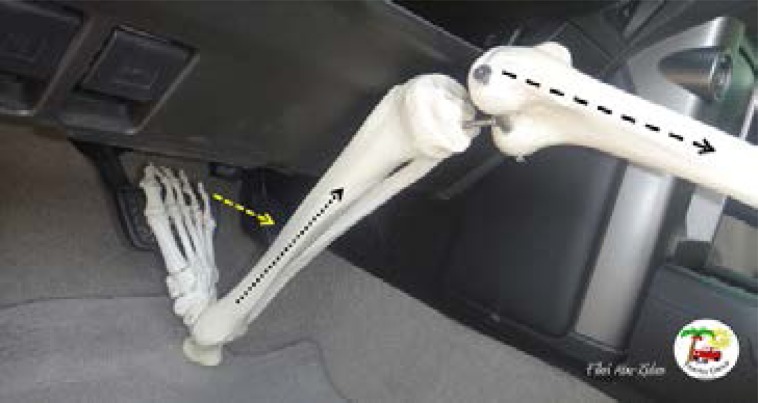
The most common mechanisms of injury in front impact collision are: the impaction of the knee with the dashboard (dashed black arrow) resulting inacetabular fracture or posterior hip dislocation; and abrupt dorsiflexion of the ankle during forceful application of the brake (dashed yellow arrow)resulting in foot and ankle fractures (dotted black arrow).

Forceful application of the brake at the time of impact would generate additional internal compressive forces (Figure 6). A recent study subjecting three cadaveric lower limbs to dorsiflexion and axial loading resulted in a medial malleolar fracture in two and an additional talar neck fracture in one subject.^19^

**Figure 7:**
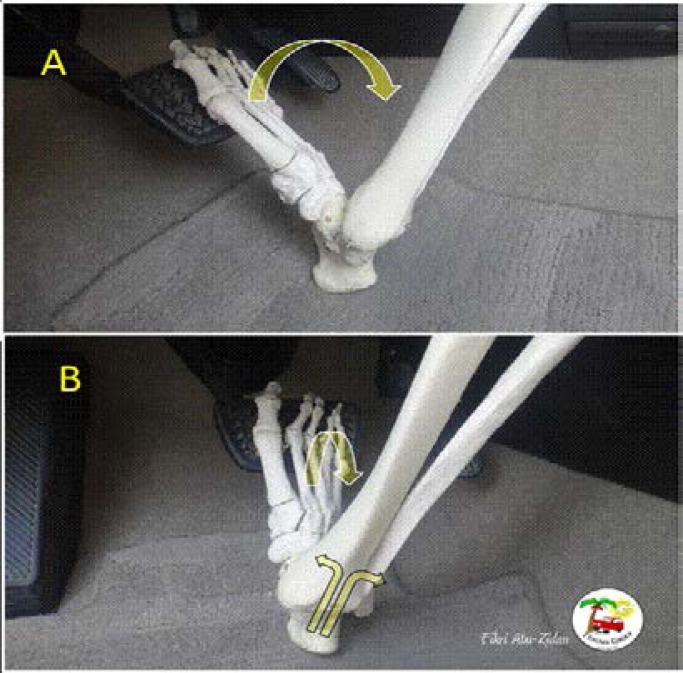
Abrupt dorsiflexion of the ankle beyond 45° in the absence of eversion may cause bilateral malleolar fractures in combination with ligament tear.

**Fig 8 (A–C):**
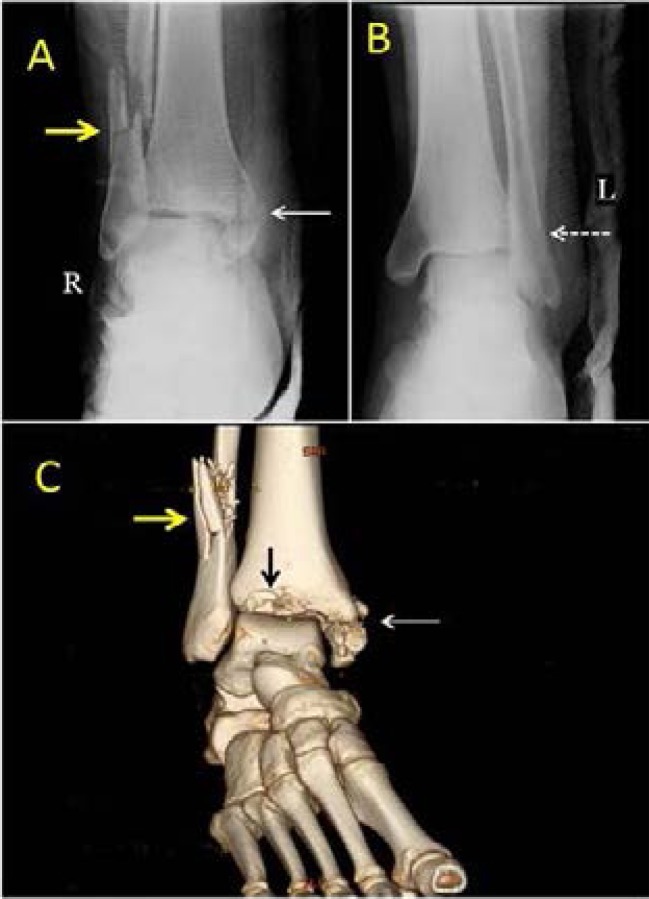
17-year-old male front seat passenger was involved in a front impact road traffic collision. The impact involved both feet. He sustained comminuted fracture of the right distal fibula (Yellow arrow), fracture of right medial malleolus (solid white arrow), and transverse fracture of the distal left fibula (white dashed arrow). CT scan with reconstruction was performed to demonstrate the behaviour of the fracture **(C)** showing a fracture of the anterior rim \of the distal tibia (black arrow) (Courtesy of Dr Ihab Abbas, Consultant orthopaedic Surgeon, Al Ain Hospital, Al Ain, UAE).

Malleolar fracture in combination with ligament avulsion may result from abrupt dorsiflexion beyond 45° in the absence of eversion^29^ (Figures 7 and 8). Another study of cadaveric specimens, which involved the use of a pendulum impactor to dynamically load the plantar surface of the foot, reported a mean force of 7.8 kn in a cohort of 12 specimens, which sustained a calcaneal fracture, versus 4.1 kn in the other 14 cadavers. A 50% probability of sustaining a calcaneal fracture secondary to a dynamic force of 6.2 kn was calculated using a logistic regression” 20 However, pre-tensioning of the Achilles tendon to simulate braking was not undertaken.

Several studies have attributed forceful braking at the time of impact to the risk of injury to the lower limb.^7^,^21^,^22^ Emergency braking could contribute 780 N to the external load produced at the pedal^3^ and generate a maximum ankle force of 10.1 kn.^22^ This mechanism is more likely to result in a tibial pylon fracture^19^ in combination with Achilles tendon rupture^22^, and less likely to produce a calcaneal fracture.^19^ This could be due to a number of reasons. Forceful application of the brake generates additional internal compressive forces through muscular activation, and pre-tensioning of the Achilles tendon, which could rupture at tensile forces ranging from 2.1 to 6.5 kn (which is generally below the 6.2 kn required for calcaneal fracture).^21^ Furthermore, plantar flexion of the foot during braking reduces the forward motion of the heel towards the toe pan, reducing the risk of calcaneal fracture.^19^ This negative association between emergency braking and calcaneal fracture is supported by the findings of Benson et al, who reported that only 35% of the drivers with calcaneal fractures had applied the brake at the point of impact.^17^

Knee to dashboard impaction, as a result of forward motion, and compartment intrusion have been identified as significant mechanisms of injury to the lower leg.^2^,^7^ Encapsulation of the knee or the foot may produce a torsional force on the tibia and fibula. This, in combination with medial perpendicular forces, due to forward motion and compartment intrusion, could result in fracture of these bones.^29^ Crandall et al reported the rate of compartment intrusion as one of the most significant predictive factors of injury to the lower limb.^7^ A higher incidence of moderate to severe foot and ankle injuries were reported in near oblique compared with direct frontal-impact collisions, and attributed to the differences in compartment intrusion.^2^

Although the use of restraint systems such as safety belts and the deployment of air bags are known to substantially reduce the severity of injury and hence mortality, the benefit to the foot and ankle is questionable. Lagares-Garcia et al concluded that the application of a safety belt did not reduce the incidence of fracture to the foot.^23^ A recent cohort study demonstrated a considerable reduction in the risk of knee-thigh-hip fractures due to the deployment of knee air bags, at the expense of an increased risk of lower leg, foot and ankle fractures. However, it should be noted that these results failed to achieve statistical significance due to the small sample size of the study.^24^

## Conclusion

Understanding the biomechanics of the lower limb injuries in frontal-impact collisions is important for their diagnosis. The most common mechanisms of injury are: the impaction of the knee with the dashboard resulting in acetabular fracture or posterior hip dislocation; and toe pan intrusion in combination with forceful application of the brake resulting in foot and ankle fractures. The probability of an occupant sustaining a significant injury to the hip is increased in taller males, and being out of position during the collision. The probability of an occupant sustaining a fracture to the foot and ankle is increased in shorter female occupants with a large overlap impact or a near oblique collision. This information may be useful in alerting clinicians to the type of lower limb injury sustained in frontal-impact collisions.
